# Degradation of Anthracycline Antitumor Compounds Catalysed by Metal Ions

**DOI:** 10.1155/MBD.1994.183

**Published:** 1994

**Authors:** Marina M. L. Fiallo, Hayet Tayeb-Bel Haj, Arlette Garnier-Suillerot

**Affiliations:** 1 Laboratoire de Chimie Bioinorganique LPCB (URA CNRS 198) Université de Paris Nord 74 rue Marcel Cachin Bobigny F - 93012 France

## Abstract

The influence of some metal ions on the degradation of anthracyclines was examined. One of the degradation products is the 7,8-dehydro-9,10-desacetyldoxorubicinone, D* (¥), usually formed by hydrolysis at slightly basic pH. D* is a lipophilic compound with no cytostatic properties. Its formation could be responsible for the lack of antitumor activity of the parent compound. The coordination of metal ions to anthracycline derivatives is required to have degradation products. Cations such as Na^+^, K^+^, or Ca^2+^ do not induce the D* formation however metals which can form stable complexes with doxorubicin afford D*. Iron(III) and copper(II) form appreciable amount of D* at slightly acidic pH. Terbium(III) forms D* but its complex is stable only at slightly basic pH. Palladium(II) which does not form D*. The influence of the coordination mode of metal ions to anthracycline on the D* formation is discussed.


### DEGRADATION OF ANTHRACYCLINE ANTITUMOR COMPOUNDS CATALYSED BY METAL IONS

Marina M.L. Fiallo*, Hayet Tayeb-Bel Haj and Arlette Garnier-Suillerot

Laboratoire de Chimie Bioinorganique, LPCB (URA CNRS 198), Université de Paris Nord, 74 rue Marcel Cachin, F - 93012 Bobigny, France

* to whom correspondence should be addressed

**¥ Abbreviations**

**D***,7,8-dehydro-9,10-desacetyldoxorubicinone; **Dox**, doxorubicin; **Dnr**, daunorubicin; **Pra**, pirarubicin; **IC50**, inhibitory concentration, the drug concentration required to inhibit 50% of cell growth; **S**, doxorubicin-sensitive K562 cells; **R**, doxorubicin-resistant K562 cells; **CD**,circular dichroïsm

**Abstract**

The influence of some metal ions on the degradation of anthracyclines was examined. One of the degradation products is the 7,8-dehydro-9,10-desacetyldoxorubicinone, D* (¥), usually formed by hydrolysis at slightly basic pH. D* is a lipophilic compound with no cytostatic properties. Its formation could be responsible for the lack of antitumor activity of the parent compound. The coordination of metal ions to anthracycline derivatives is required to have degradation products. Cations such as $Na^+$, $K^+$, or $Ca^{2+}$ do not induce the D* formation however metals which can form stable complexes with doxorubicin afford D*. Iron(III) and copper(II) form appreciable amount of D* at slightly acidic pH. Terbium(III) forms D* but its complex is stable only at slightly basic pH. Palladium(II) which does not form D*. The influence of the coordination mode of metal ions to anthracycline on the D* formation is discussed.

### Introduction

Degradation of antitumor compounds is a common problem for pharmacologists.[1] The efficacy of an antitumor drug is related to the intracellular drug concentration as well as to the rate of cell uptake.[2] Anthracycline antibiotics, Doxorubicin (I) and Daunorubicin (I')(Chart 1) are benzanthraquinone drugs, which are useful in the treatement of several type of human malignancies.[3] Anthracycline are known to bind different metal cations and to form several complexes with, usually, high stability constants.[4]

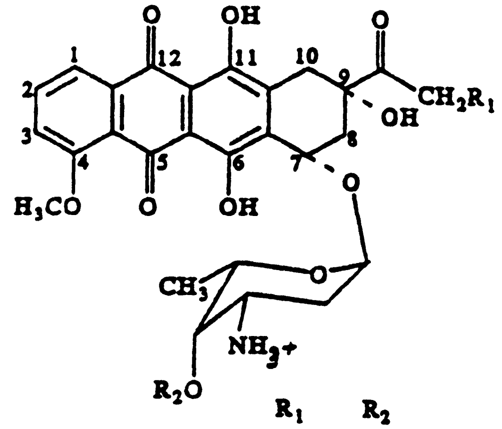

| | | $R_1$ | $R_2$ |
|---|---|---|---|
| I | Doxorubicin | OH | H |
| I' | Daunorubicin | H | H |
| I'' | Pirarubicin | OH |  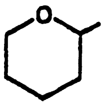 |

Chart 1

Complexation of these biological active molecules with metal ions has been examined in order i) to reduce the toxic effects such as dose-dependent cardiotoxicity;[5] ii) to improve the antitumor properties;[6] iii) to reproduce some in vivo mechanisms.[7]

In order to determine the relationships between intracellular drug concentration and the cytotoxic activity (as well as the degree of resistance) we were interested in the anthracycline degradation in cell culture.

One of the degradation products of anthracycline, namely 7,8-dehydro-9,10-desacetyl-doxorubicinone, D* (II)(Chart 2), has been identified[8,9] however its biological effects have been neglected by pharmacologists. In a previous report we have shown that this derivative, which is detected in cells following incubation with doxorubicin, is formed in the culture medium.[10]

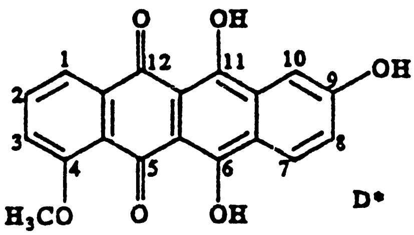

D* II

Chart 2

We also detected the presence of D* when studying the interactions of cells with the iron complexes of doxorubicin and pirarubicin; in this case the degradation product is not formed in the medium but it is already present in the metal complex solution.[11] These outcomes prompted us to further investigate whether other metal ions can form D* giving us the opportunity to discuss about the chemical requirements necessary to have D* formation.

### Results

The metal complexes of anthracycline were prepared following the published procedures[12-15] and dissociated by adding hydrochloric acid (0.2 M). The dissociation step usually required less than one hour except for the Pd(II) complexes. We verified that the strongly acidic pH (pH<1) was not responsible of the degradation of anthracyclines. Control experiments were done with the free anthracycline and D* was not formed in the experimental conditions employed without metal ion.

*Iron(III)-anthracycline systems*

Iron(III) forms with doxorubicin (and pirarubicin) a complex in which three anthracycline are bound to the metal ion following an octahedral arrangement, $[Fe(Dox)_3]$ (**III**) and $[Fe(Pra)_3]$ (**IV**).[16] Magnetic susceptibility measurements indicate that the complexes thus obtained are in high spin-form suggesting that six oxygens are bound to the iron.[12] The anthracyclines act as bidentate ligands through the C12-carbonyl and the C11-phenolate oxygens forming a six membered chelate ring.[17] Usually the iron complexes are prepared starting from iron(II), in the form of Mohr's salt as source of iron(III), following air-oxidation to iron(III). This method is generally considered the best way to prepare these complexes and to avoid formation of iron hydroxides.[18] The metal ion can be added either to an anthracycline buffer solution at pH 7 (method i) or to an unbuffered solution and the subsequent addition of the stoichiometric amount of NaOH necessary to deprotonate once the anthracycline (ii). Both reactions are very fast and depending on the procedures used, two different CD spectra were obtained. In buffer solution (method i) and also in the presence of an excess of iron, the CD spectrum is prevalently characterized by a strong positive band centred at 630 nm (Fig. 1); this band is typical of the iron complexes of daunorubicin derivatives[12,19] and characterizes what will be hereafter labelled a D-type spectrum. 24 hours later, after dissociation of the complex, we detected the presence of D* in an amount ranging from 0 to 10% of the initial anthracycline concentration.

If the metal complexes are prepared in water with the subsequent addition of NaOH, 24 hours later the amount of D* raised to 25-30%: the CD spectrum did not evolve with time and still exhibited two positive bands at 500 and 605 nm, characteristics of an A-type spectrum (Fig. 1).[12] The cytostatic properties of the A-type complex are drastically reduced, particularly in the case of doxorubicin-resistant cells (IC50 [R]=3000 nM compared to 200 nM for Dox).

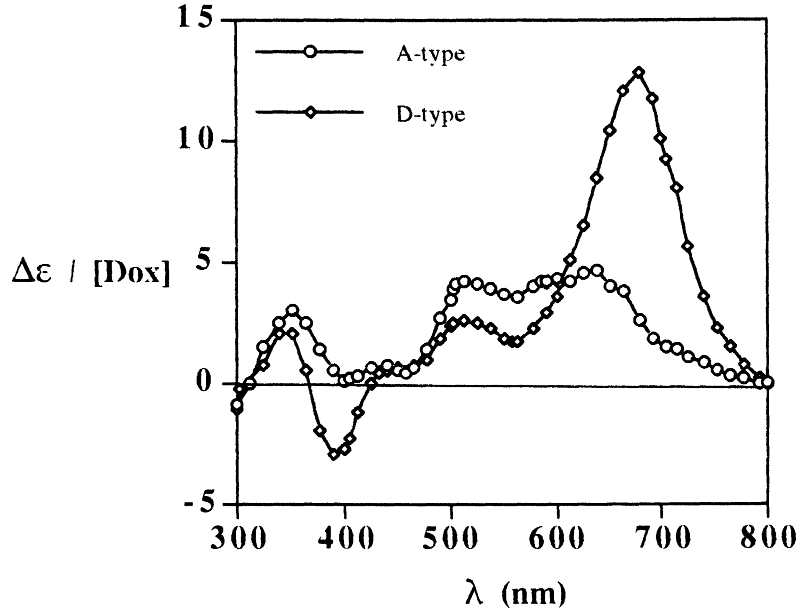

**Figure 1.** CD spectra of the 1:3 Fe(III)/Dox complex, in the A-type and in the D-type forms. Experimental conditions: [Dox]= $5x10^{-4}$ M; [KCl] = 0.01 M; pH 6.0.

This complex is formed in water at pH around 5-6; at these values the hydrolytic pathway to get D* is discouragead.[8] In fact in the same conditions, free doxorubicin did not yield D*.

*Copper(II)-doxorubicin systems*

Copper(II) is known to form with doxorubicin two complexes depending on the pH and on the stoichiometric ratio.[12,20] At pH 6.5 a first complex is formed: two anthracyclines are bound to the metal ion through the oxygens of one phenolate and one carbonyl functions, $[Cu(Dox)_2]$ (**V**), with no particularly preference for one position.[21] At pH higher then 7, the prevalent species is a 1:1 complex, probably with a polymeric structure, $[Cu\text{-}Dox]_n$ (**VI**). The two complexes were prepared as described[13] and their spectroscopic properties were checked. The dissociation of the two complexes was very fast: in less than 30 minutes an orange solution was recovered indicating the presence of free doxorubicin. Two hours after their preparation, we checked the cytostatic properties as well as the doxorubicin degradation of the complex **V**. We found the same IC50 (4 nM for [S] cells) that for the free drug and no detectable D*. 24 hours later we checked again: the metal complex was inefficient against tumour cells at the same concentration of free doxorubicin (IC50[S]>10nM) and an amount of D* ranging from 8 to 10% was detected, with no difference in the experimental conditions used.

*Terbium(III)-anthracycline systems*

At pH 7.2 terbium(III) binds two molecules of anthracycline, forminq $[Tb(Dox)_2]$ (**VII**).[22] The coordination has been established also by NMR spectra to occur through the oxygens at C11 and C12.[14,23] The CD spectrum of complex **VII** exhibits positive bands at 490, 530 and 560 nm and one negative at 595 nm (Fig. 2).

Dissociation of **VII** was very fast and all the initial doxorubicin was recovered (IC50[S]=5.5 nM). This complex evolved with time: after three weeks the new CD spectrum exhibited positive bands at 540 and 570 nm. We can accelerate the formation of this second species, **VIII**, raising the reactionnel pH to 8. In these conditions only three days are required. The dissociation of **VIII** was very slow and we recovered only the 50% of the initial amount of doxorubicin, the remainder being degraded to D*.

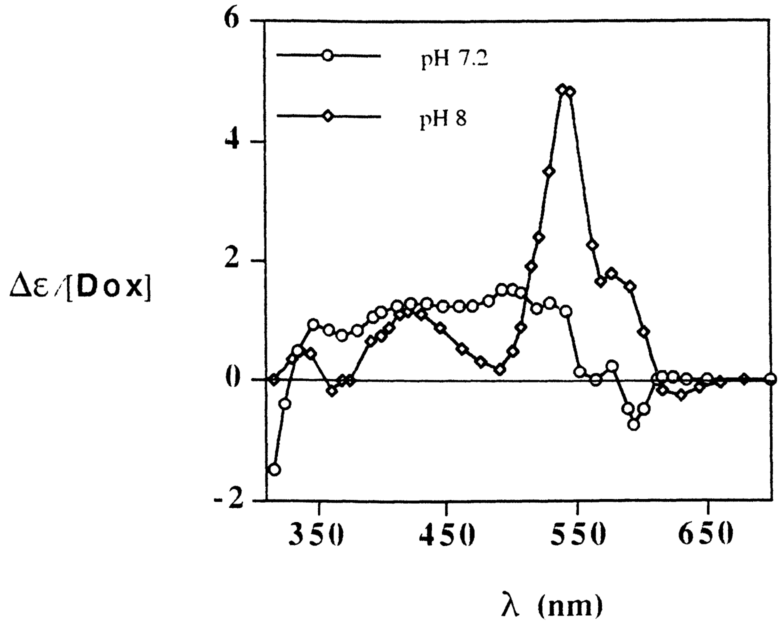

**Figure 2**. CD spectra of the 1:2 Tb(III)/Dox complex, after 2 hour (pH 7.2) and after 3 days (pH 8). Experimental conditions: [Dox] = $1\times10^{-4}$ M; [KCl] = 0.01 M

*Palladium(II)-doxorubicin systems*

Palladium(II) reacts very slowly with doxorubicin; the reaction can take place only in slightly acidic pH.[15] Palladium(II) forms with the doxorubicin two complexes. In the first one, **IX**. two drug molecules are bound to one metal ion forming [Pd(Dox)$_2$] whereas the formation of the second species, **X**, occurs to a 1:1 molar ratio of Pd(II) per doxorubicin. Coordination of palladium(II) to the anthracycline would occur at the C12-carbonyl and the C11-phenolate oxygens and to the amino group of daunosamine.[24] In particularly in the complex **IX**, one doxorubicin is bound through the oxygens at C11 and C12 and a second one by the NH$_2$ group. The complex **X**, [Pd-Dox]$_2$, is a dimer where the two coordinative positions still free in **IX** are occupied by another Pd(II) ion. The coordination to the amino group has been confirmed also by NMR spectra.[25]

As palladium(II) is inert, dissociation of **IX** and **X** required more time (24 hours) and stronger conditions (1.2 M HCl) than all other complexes tested. We checked the cytostatic properties of the two complexes: we found that the complexation to Pd(II) ions (IC50[R]=290 nM) did not modify the inhibitory effect on the cell proliferation of free doxorubicin (IC50[R]= 200 nM) and no D* was detected.

### Discussion

The instability of anthracycline derivatives in aqueous solution and in cell culture media has been recognised by several investigators.

Among them Beijnen *et al.*[8,9,26] have identified the anthracycline degradation products formed in acidic media (pH<4) or slightly basic pH media (pH~8). They showed that degradation of doxorubicin at pH higher than 7 yielded mainly a pink coloured aglycone in which full aromatization of the ring A occurred. This compound, identified as D*, can be also formed at strongly acidic pH (pH<1)[27] and by photodegradation.[28] Structural analogies of the doxorubicin with corticosteroids, i.e. hydrocortisone, suggested a possible mechanism of the D* formation at basic pH.[8] Corticosteroids possess a C17 $\alpha$-ketol side chain[29] analogue to the C9 $\alpha$-ketol substituent in doxorubicin which could constitute an extra protonation site. Reversible enolization and ionization of this function should be possible at basic pH: the determination of the corresponding pKa would be hampered by overlap with the deprotonation of the phenolic group. In analogy with the degradation of corticosteroids, the enolization step is assumed to be the first step of the degradation reaction of doxorubicin. The enolate anion arising from keto/enol tautomerization and deprotonation may be involved in a tautomeric equilibrium with its 13-ol-14-aldehyde derivative. A concertated mechanism entraining the cleavage of the C9 side chain and the aminosugar moiety yields D*. The high stability of the resulting degradation product is the driving force of the full aromatization of the A ring after cleavage of the C9 side chain.

D*, a lipophilic compound, enters in cells very rapidly but unfortunately it has no cytostatic properties:[11] its formation could explain the reduced antiproliferative activity observed in the case of degradated anthracycline.[10]

An influence of metal ions on the D* degradation was already proposed[26] but not verified. In the case of the Tb(III) complexes, the slightly basic pH at which this complex is formed could be considered as responsible of the doxorubicin degradation. It has to be observed that at the same pH value, free doxorubicin is more degrated (50% in 1 day) than if complexed to terbium(III) ions. It means that in this case the metal ion seems to have a protecting role against the D* formation.

However from our results the formation of D* seems to be related to the different coordination mode of the metal ions. In fact coordination at the C5-C6 site, which has been established for Cu(II)[21] and Tb(III)[14] seems to prevent D* formation more than the coordination at the C11-C12 site. The anomalous case of terbium(III) has been related to the high value of the reactionnel pH. The C11-C12 position has probably a positive effect on the enol formation, first step of the degradation pathway.

In addition under slightly basic conditions the rate of the glycosidic bond cleavage is strongly dependent on the electronic structure of the aglycone part[8] while at acidic pH the structural modifications in the sugar moiety are more important.[26]

Palladium(II) is coordinated to doxorubicin also by the amino group of the daunosamine and, in this case D* has been never observed.

This important difference in the coordination mode of metal ion seems to play a role in D* formation. This fact could be related to the cleavage of the glycosidic bond which is a crucial step in the concertated mechanism yielding the D* formation.

From a recent paper[31] we can suggest a possible explanation for the D* formation in the iron complexes. Reexamining the structure of [Fe(Dnr)$_3$] by EPR, EXAFS, and Mössbauer techniques, Matzanke et al. observed that at the 1:3 [Fe]/[Dnr] ratio the complex exists only in a polymeric form. To justify the anisotropy of this complex two "speculative" models of polymer were proposed: in the first one [Fe(Dnr)$_3$] octahedra are attached to each other by Dnr-Dnr stacking forming a three dimensional polymeric structure . In the second case a linear polymer would be composed of planes of two Dnr molecules binding Fe(III) ions with the oxygens at C11 and C12. The axial positions would be occupied by OH groups bridging two planes. Planes of Dnr dimers would be randomly distributed in the linear polymer with in both cases the amino group of the daunorubicin involved in the stacking of the anthracyclines.

Making the hypothesis that the structure of the [Fe(Dox)3] complex exhibiting a D-type CD spectrum is similar to that of the [Fe(Dnr)3] we can suggest that the involvment of the amino group in the Dox stacking prevent D* formatio as it was observed in the case of the palladium complex. Obviously these hypothesis require to be verified by the analysis of other complexes of anthracycline, which are actually under study.

**Acknowledgements**. This investigation was supported by Université Paris Nord and Centre National de la Recherche Scientifique

**References**

1. Pavlik, E.J., Kenady, D.E., van Nagell jr., J.R., Hanson, M.B., Donaldson, E.S., Casper, S., Garrett, D., Smith, D., Keaton, K., and Flanigan, R.C. *Cancer Invest.* **1984**,2,449
2. Frezard, F. and Garnier-Suillerot, A. *Eur. J. Biochem.* **1991**,*196*,483
3. Arcamone, F. in "*Metabolism of Xenobiotics*" (Garrod, J.W., Oelschäger, H. and Caldwell, J. eds.) Taylor & Francis, London **1988**, pp. 115
4. Garnier-Suillerot, A. in "*Anthracycline and Anthracenedione-based Anticancer Agants*" Lown, J.W. ed.) Elsevier N.Y. **1988**, pp.129
5. Gosalvez, M., Bianco, M.F., Vivero, C. and Valles, F. *Eur. J. Cancer* **1978**,*14*,1185; Bono, V.H. in "*Anthracyclines: Current Status and New Develpments*" (Crooke, S.T. and Reich, S.D. eds) Academic press N.Y. **1980**,pp.315
6. Moustatih, A., Fiallo, M.M.L., and Garnier-Suillerot, A. *J. Med. Chem.* **1989**,32,336; Pasini, A. *Inorg. Chim. Acta* **1987**,*137*,123
7. Canada, R.G., Saway, W., and Thompson, E. *Biochem. Biophys. Res. Comm.* **1988**,*151*,679; Canada, R.G. *Anal. Chim. Acta* **1988**,205,77
8. Beijnen, J.H., van der Houwen, O.A.G.J., and Underberg, W.J.M. *Int. J. Pharm.* **1986**,32,123
9. Beijnen, J.H., Potman, R.P., van Ooijen, R.D., Driebergen, R.J., Voskuilen, M.C.H., Renema, J., and Underberg, W.J.M. *Int. J. Pharm.* **1987**,34,247

10. Fiallo, M., Laigle, A., Borrel, M.-N., and Garnier-Suillerot, A. *Biochem. Pharmacol.* 1993,45,659

11. Fiallo, M.M.L., Laigle, A., Garnier-Suillerot, A., Amirand, C., Ballini, J.-P., Chinski, L., Dusquesne, M., Jollés, B., Sureau, F., Turpin, P.-Y., and Vigny, P. *Biochim. Biophys. Acta* 1993,1177,236

12. Beraldo, H., Garnier-Suillerot, A., Tosi, L., and Lavelle, F. *Biochemistry* 1985,24,284

13. Beraldo, H., Garnier-Suillerot, A., and Tosi, L. *Inorg. Chem.* 1983,22,4117

14. Tayeb-Bel Haj, H., Fiallo, M.M.L., Garnier-Suillerot, A., Kiss, T., and Kozlowski, H. in preparation

15. Fiallo, M.M.L. and Garnier-Suillerot, A. *Biochemistry* 1986,25,924

16. Myers, C.E., Gianni, L., Simone, C.B., Klecker, R., and Greene, R. *Biochemistry* 1982,21,1707

17. Muindi, J.R.F., Sinha, B.K., Gianni, L., and Myers, C.E. *FEBS Lett.* 1984,172,226

18. Gelvan, D. and Samuni, A. *Cancer Res.* 1988,48,5645

19. Fiallo, M.M.L. and Garnier-Suillerot, A. *Biochim. Biophys. Acta* 1985,840,91

20. Kiraly, R. and Martin, R.B. *Inorg. Chim. Acta* 1982,67,12

21. Fiallo, M.M.L. and Garnier-Suillerot, A. *J. Inorg. Biochem.* 1987,31,43

22. Mariam, Y.H., Wells, W., and Wright, B. *J. Sol. Chem.* 1984,13,259; Canada, R.G. and Carpentier, R.G. *Biochim. Biophys. Acta* 1991,1073,136

23. Mc Lennan, I.J., Lenkinski, R.E., and Yanuka, Y. *J. Am. Chem. Soc.* 1985,106,6905

24. Arcamone, F., Cassinelli, G., Orezzi, P., Franceschi, G., and Mondelli, R. *J. Am. Chem. Soc.* 1964,86,5335

25. Allman, T. and Lenkinski, R.E. *J. Inorg. Biochem.* 1987,30,35

26. Beijnen, J.H., van der Nat, J.M., Labadie, R.P., and Underberg, W.J.M. *Anticancer Res.* 1986,6,39

27. Arcamone, F., Franceschi, G., Orezzi, P., Cassinelli, G., Barbieri, W. and Mondelli, R. *J. Am. Chem. Soc.* 1964,86,5334

28. Gray, P.J. and Phillips, D.R. *Photochem. Photobiol.* 1981,33,297; Williams, B.A. and Tritton, T.R. *Photochem. Photobiol.* 1981,34,131

29. Hansen, J. and Bundgaard, H. *Arch. Pharm. Chem. Sci. Ed.* 1979,7,135; Johnson, D.M. *J. Org. Chem.* 1982,47,198

30. Beijnen, J.H., Wiese, G., and Underberg, W.J.M. *Pharm. Weekbl. Sci. Ed.* 1985,7,109

31. Matzanke, B.F., Trautwein, A.X., Winkler, H. Hermes, C., Nolting, H.-F., Barbieri, R. and Russo, U. *Eur. J. Biochem.* 1992,207,745

**Received: September 7, 1993**

